# The Influence of Biofilms on Carbapenem Susceptibility and Patient Outcome in Device Associated *K. pneumoniae* Infections: Insights Into Phenotype vs Genome-Wide Analysis and Correlation

**DOI:** 10.3389/fmicb.2020.591679

**Published:** 2020-12-14

**Authors:** Naveen Kumar Devanga Ragupathi, Dhiviya Prabaa Muthuirulandi Sethuvel, Hariharan Triplicane Dwarakanathan, Dhivya Murugan, Yamini Umashankar, Peter N. Monk, Esther Karunakaran, Balaji Veeraraghavan

**Affiliations:** ^1^Sheffield Collaboratorium for Antimicrobial Resistance and Biofilms (SCARAB), The University of Sheffield, Sheffield, United Kingdom; ^2^Department of Chemical and Biological Engineering, The University of Sheffield, Sheffield, United Kingdom; ^3^Department of Clinical Microbiology, Christian Medical College, Vellore, India; ^4^Department of Orthopaedics, Christian Medical College, Vellore, India; ^5^Department of Infection, Immunity and Cardiovascular Disease, The University of Sheffield, Sheffield, United Kingdom

**Keywords:** *K. pneumoniae*, biofilm, genome, carbapenem resistance, molecular epidemiology

## Abstract

*Klebsiella pneumoniae* is one of the leading causes of nosocomial infections. Carbapenem-resistant *K. pneumoniae* are on the rise globally. The biofilm forming ability of *K. pneumoni*ae further complicates patient management. There is still a knowledge gap on the association of biofilm formation with patient outcome and carbapenem susceptibility, which is investigated in present study. *K. pneumoniae* isolates from patients admitted in critical care units with catheters and ventilators were included. *K. pneumoniae* (*n* = 72) were subjected to 96-well plate biofilm formation assay followed by MBEC assay for subset of strong biofilm formers. Whole genome sequencing and a core genome phylogenetic analysis in comparison with global isolates were performed. Phenotypic analyses showed a positive correlation between biofilm formation and carbapenem resistance. Planktonic cells observed to be susceptible *in vitro* exhibited higher MICs in biofilm structure, hence MICs cannot be extrapolated for treatment. The biofilm forming ability had a significant association with morbidity/mortality. Infections by stronger biofilm forming pathogens significantly (*p* < 0.05) resulted in fewer “average days alive” for the patient (3.33 days) in comparison to those negative for biofilms (11.33 days). Phylogenetic analysis including global isolates revealed clear association of sequence types with genes for biofilm formation and carbapenem resistance. Known hypervirulent clone-ST23 with *wcaG*, *magA*, *rmpA*, *rmpA2*, and *wzc* with lack of mutation for hyper-capsulation might be poor biofilm formers. ST15, ST16, ST307, and ST258 (reported global high-risk clones) were *wcaJ* negative indicating the high potential of biofilm forming capacity. Genes *wabG* and *treC* for CPS, *bcsA* and *pgaC* for adhesins, *luxS* for quorum sensing were common in all clades in addition to genes for aerobactin (*iutA*), allantoin (*allS*), type I and III fimbriae (*fimA*, *fimH*, and *mrkD*) and pili (*pilQ* and *ecpA*). This study is the first of its kind to compare genetic features of antimicrobial resistance with a spectrum covering most of the genetic factors for *K. pneumoniae* biofilm. These results highlight the importance of biofilm screening to effectively manage nosocomial infections by *K. pneumoniae*. Further, data obtained on epidemiology and associations of biofilm and resistance genetic factors will serve to enhance our understanding on biofilm mechanisms in *K. pneumoniae*.

## Introduction

*Klebsiella pneumoniae* remains one of the leading causes of nosocomial infections and declared by WHO as a “priority pathogen.” In recent times, carbapenem-resistant *K. pneumoniae* (CRKP) have been on the rise, leading to multi-drug resistance (MDR) and limiting treatment options. Emergence of MDR *K. pneumoniae* is a cause for current concern in many countries worldwide with a mortality rate of ∼42% for CRKP (Xu et al., 2017).

*Klebsiella pneumoniae* are also known to cause biofilm-mediated infections in most of the hospitalized patients; this, in addition to carbapenem resistance, complicates the treatment. Biofilms are complex mono or polymicrobial structures, result in persistent prolonged infections, difficult to treat and clear *in vivo*. Most of them result from indwelling medical devices such as catheters and ventilators, expected to be coated with host cellular factors *in situ*, leading to nosocomial infections (Murphy and Clegg, 2012). Biofilm-mediated infections have remained under-researched for decades and their actual significance in influencing the outcome of antimicrobial therapy is yet to be fully understood.

Some of the virulence factors of *K. pneumoniae* include capsule polysaccharide, lipopolysaccharide, type 1 and type three fimbriae, outer membrane proteins and determinants for iron acquisition and nitrogen source usage (Murphy and Clegg, 2012; Li et al., 2014). These virulence factors are known to be essential for the pathogen to evade host immune system and for successful biofilm formation (Murphy and Clegg, 2012; Li et al., 2014). The biofilm-forming phenomenon in *K. pneumoniae* was first described in 1988 (LeChevallier et al., 1988).

Biofilm formation mechanisms in clinical *K. pneumoniae* is reported to be mediated by a series of genes, including allantoin (*allS*), aerobactin (*iutA*), type I (*fimA* and *fimH*) and type III fimbriae (*mrkA* and *mrkD*), polysaccharides and adhesins (*pgaA*, *pgaB*, *pgaC*, and *bcsA*), capsular polysaccharide (CPS) (*wzc, cpsD, treC, wcaG, wabG, rmpA/A2, magA, k2a*, and *wzyk2*), quorum sensing (QS) (*luxS*) and colonic acid (*wcaJ*) (Wu et al., 2011; Alcántar-Curiel et al., 2013; Zheng et al., 2018; Pal et al., 2019; Chen et al., 2020; Hasani et al., 2020). Though these genetic factors were individually studied, a collective approach on their analysis in a set of clinical isolates is still lacking.

Antimicrobial resistance (AMR) is a global problem but very limited data are available on biofilm formation and its association with AMR among clinical isolates of *K. pneumoniae*. This study tries to address the gap in understanding the effect and association of *K. pneumoniae* biofilm formation on AMR in nosocomial infections. In addition, the study provides information on the epidemiology of *K. pneumoniae* on their genetic make-up for biofilm formation and which possibly enables the success of high-risk clones globally.

## Materials and Methods

### Study Isolates

Isolates with significance from invasive samples in culture were only considered for inclusion in the study. Accordingly, a total of 72 *K. pneumoniae* non-duplicate isolates meeting the nosocomial infections criteria (Garner et al., 1988; Ducel et al., 2002), previously obtained from clinical samples blood (*n* = 55) and endotracheal aspirates (*n* = 17) from patients in ICU/high-dependency units at the Christian Medical College, Vellore, India between 2018–2019 were selected. These isolates were subjected to antimicrobial susceptibility and biofilm formation analyses, and whole-genome sequencing (WGS).

### Antimicrobial Susceptibility Testing

#### Disc Diffusion

Antimicrobial susceptibility testing was performed by the Kirby-Bauer method with amikacin (30 μg), chloramphenicol (30 μg), tetracycline (30 μg), gentamycin (10 μg), ciprofloxacin (5 μg), cefotaxime (30 μg), cefoxitin (30 μg), ceftazidime (30 μg), cefpodoxime (10 μg), piperacilllin-tazobactam (100/10 μg), cefoperazone-sulbactam (75/30), netilmicin (30 μg), imipenem (10 μg), meropenem (10 μg), and tigecycline (15 μg) according to guidelines suggested by CLSI M100-S29, 2019 (CLSI). Quality control strains used were *Escherichia coli* ATCC 25922 for all antibiotics concurrently in all the batches. Tigecycline results were interpreted according to FDA criteria.

#### Minimum Inhibitory Concentration Testing

Minimum Inhibitory Concentration Testing (MIC) tests were performed for meropenem by the broth microdilution method. *E. coli* ATCC 25922 was used as quality control strain for MIC determination with the expected range of 0.008 – 0.06 μg/ml for meropenem. The interpretive criterion provided by CLSI Clinical and Laboratory Standards Institute, 2019 for susceptible, intermediate and resistant strains were ≤1, 2, and ≥4 μg/ml for meropenem.

### Screening for Biofilm Formation

#### Biofilm Screening Assay

The protocol used was slightly modified from the method described by Di Domenico et al. (2016). About 5–10 colonies from fresh culture were inoculated in a 10 ml LB broth and incubated for 12–18 h at 37°C. Optical density (OD) was measured in a spectrophotometer (Shimadzu, Kyoto, Japan) at 625 nm and 0.05 OD cells were prepared by dilution in Mueller-Hinton broth (MHB) broth (1% glucose). 150 μl of prepared cells were inoculated into each well on a 96 well plate and incubated at 37°C for 24 h. After incubation, the medium was removed and the biofilm was washed with 200 μl distilled water. For staining, 200 μl of 0.1% (w/v) crystal violet stain was added and incubated for 10 min at RT. Wells were washed and destained using 200 μl of 33% (v/v) glacial acetic acid followed by incubation for 5 min at RT. OD was read at 570 nm. The assay was performed in triplicates and a blank well containing only growth medium without cells were used as negative control for the calculation of biofilm formation efficiency. Mean and SD was calculated for statistical significance.

The OD_570_ values were used to compare and classify biofilm production semi-quantitatively. Accordingly, the cut-off OD (ODc) was calculated mean OD of negative control with three standard deviations. Biofilm production was classified as: OD < ODc = poor biofilm producer; ODc < OD ≤ 2 × ODc = weak biofilm producer; 2 × ODc < OD < 4 × ODc = moderate biofilm producer; and OD ≥ 4 × ODc = strong biofilm producer.

#### Minimum Biofilm Eradication Concentration Estimation Assay

An Minimum Biofilm Eradication Concentration (MBEC) assay was performed for the study isolates using the method previously described by Chen P. et al. (2014) with slight modifications. Briefly, all isolates were grown in cation-adjusted MHB with 1% glucose at 37°C overnight. Cultures were then adjusted for 0.05 OD using MHB and 150 μL of this adjusted inoculum was added to all the wells (except 1 well – sterility control) in a 96-well MBEC Assay^®^ bottom plate (Innovotech, AB, Canada). The MBEC inoculator plate was then inserted and incubated at 37°C for 24 h to allow biofilm formation. After the production of biofilm in the inoculator plate, four pegs from it were taken using a sterile tool and tested for the density of biofilm formed.

For this, the removed pegs were put in a fresh 96-well plate (A1–A4) with 200 μl TSB medium with 1% Tween20 (rich medium) and sonicated in high power for 10 min. After sonication, 20 μl of inoculum from the suspension (planktonic cells) in the MBEC bottom plate (any four random wells) were inoculated in four wells (A5–A8) with 180 μl of rich medium. The suspensions were then serially diluted up to 10^–8^ dilutions, and 10 μl was plated on LB agar followed by incubation at 37°C overnight to record the CFU/ml.

The rest of the inoculator plate with biofilm was inoculated into a 96-well plate containing various concentrations of the antibiotic in 200 μl MHB in duplicates. The MBEC plate setup with antibiotics was incubated at 37°C for 24 h. Following incubation, the inoculator plate was removed and washed with sterile distilled water for 1 min and introduced in a 96-well bottom plate with 200 μl of rich medium. The plate was sonicated for 10 min at high power. Released biofilm cells (sonicated) and planktonic cells from MBEC bottom plate was serially diluted up to 10^–4^ dilutions. 10 μl was plated in LB agar followed by incubation at 37°C overnight. The LB plates were recorded for CFU/ml and the MBEC values were obtained.

### Whole Genome Sequencing

Six isolates were sequenced for analysis of carbapenem resistance and other genetic factors involved. Genomic DNA was extracted with QIAamp DNA mini kit (Qiagen, Hilden, Germany). Whole genome sequencing (WGS) was performed using Ion Torrent (PGM) sequencer with 400-bp read chemistry (Life Technologies) according to the manufacturer’s instructions. The data was assembled *de novo* using AssemblerSPAdes v5.0.0.0 embedded in Torrent suite server version 5.0.3. The sequence annotation was performed in NCBI Prokaryotic Genomes Automatic Annotation Pipeline (PGAAP)^1^. Downstream analysis was done using the Center for Genomic Epidemiology (CGE) server^2^.

### Genome-Wide Association and Phylogenetic Analysis

In addition to the six isolates sequenced in this study, complete genome sequences of 473 isolates (451 *K. pneumoniae* and 22 *K. quasipneumoniae*) with reported biofilm genotypes were selected and downloaded from NCBI for a global comparison of the study isolates. Search terms used were “*K. pneumoniae* + biofilm;” “*Klebsiella quasipneumoniae* + biofilm.” Accordingly, *K. pneumoniae* and *K. quasipneumoniae* were analyzed separately for their phylogenetic relations. Fasta sequences were used to call core SNPs using Snippy v4.4.0^3^ and recombinations were removed using Gubbins v2.0.0 (Croucher et al., 2014). RAxML program was used to build the phylogenetic trees using the clean core SNP alignments generated from Gubbins.

Obtained 473 fasta sequences were annotated using Prokka v1.14.6 for further analysis (Seemann, 2014). The isolates were analyzed using Roary v3.11.2 (Page et al., 2015) with Mafft v7.467 to identify the core genes involved among the selected population of *K. pneumoniae*, which was used to study the genes involved in biofilm production. Sequences were then analyzed using ABRicate v0.8.7 for their AMR genes and plasmids; MLST was identified using mlst v 2.18.0 algorithm^4^. Country-wise distribution of carbapenemases and *mcr* genes and their association with sequence types (STs) in 454 *K. pneumoniae* including study isolates was mapped using mapchart.net^5^. Individual biofilm gene sequences were retrieved using a blast algorithm, which were further subjected to Snippy for generating a core alignment and the tree was built using RaxML. All trees generated in the study were visualized using iTOL v 5.5.1. Scoary v1.6.16 was used for genotypic association analysis with parameters “-c I EPW -p 0.1 0.05–collapse” by providing core genome alignment tree generated by Roary (Brynildsrud et al., 2016).

This Whole Genome Shotgun project has been deposited at GenBank under the accession numbers JAAMFF00000000, JAALJL00000000, JAALJK00000000, JAAMFE00000000, JAAMFD00000000, and JAALJJ00000000.

### Statistical Analysis

Clinical characteristics were recorded in Microsoft Excel 2016 (Roselle, IL, United States) and analyzed for significance in SPSS 16.0 using *t*-test. Logarithmic growth values of meropenem treated biofilms were calculated as mean ± SD of triplicate values. GraphPad Prism v8.2.0 was used to generate the MBEC graph. Correlation of AMR and biofilm virulence genes were recorded as significant only if Benjamini-Hochberg’s *p*-value were *p* < 0.05.

## Results

### Antimicrobial Resistance

Among the 72 isolates tested from patients under critical care, ∼20% had carbapenem susceptibility, followed by minocycline and tigecycline, with 30 and 45% susceptibilities, respectively. In contrast, most of other tested antimicrobials had <20% susceptibility (Supplementary Table 1).

### Association of Carbapenem Resistance and Clinical Outcome With Biofilm Formation Efficiency

The biofilm formation assay revealed that out of 72 isolates tested, 27.78% (*n* = 20) were strong biofilm producers with a positive correlation with carbapenem resistance compared to moderate (22.22%), weak (19.44%) and negative (30.56%) biofilm formers (Table 1).

**TABLE 1:** Distribution of carbapenem resistance, mortality and average days alive among patients with strong, moderate, weak, and negative biofilm forming *K. pneumoniae* infections.

(*n* = 72)	% of isolates	% Carbapenem Resistance	% Of patients with negative outcome (expired)	Average no. of days alive between culture positive and expiry	% Of patients with positive outcome (discharged)
Strong biofilm (*n* = 20)	27.78	90*	38.24*	3.33*	20.59*
Moderate biofilm (*n* = 16)	22.22	81.25*	26.47*	3.875*	17.65*
Weak biofilm (*n* = 14)	19.44	85.71*	17.65	4.83*	17.65*
Negative (*n* = 22)	30.56	68.18	17.65	11.33	44.12

*P < 0.0001 – Significant difference when compared to the negative biofilm formers.

Comparison of mortality and length of hospital stay from the onset of infection revealed the average “days alive” of a patient for weak biofilm was 4.83, moderate biofilm was 3.875, strong biofilm forming pathogens was 3.33. This was significantly low (*p* < 0.05), when compared to 11.33 days for patients with isolates negative for biofilm production (Table 1). In addition, the number of patients expired were significantly higher (38.2%) in infections with strong biofilm forming *K. pneumoniae*, in comparison with biofilm negative *K. pneumoniae* (17.65%) (Supplementary Table 1).

**FIGURE 1:**
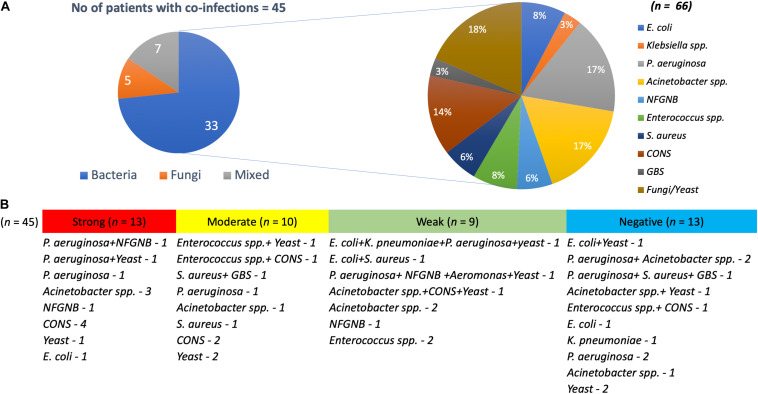
Pathogens co-infecting patients with *K. pneumoniae*. **(A)** Of 45 *K. pneumoniae* infections, 33 were co-infected with bacteria, five with yeast and seven were mixed infections. Also depicted is the number of each clinical pathogen among the bacterial co-infected population. **(B)** Numbers on each category with classification under strong, moderate, weak, and negative biofilm forming *K. pneumoniae*.

Interestingly, around 62% (*n* = 45) of the patients with *K. pneumoniae* were found co-infected with bacterial and yeast pathogens, with both monomicrobial and polymicrobial co-infections (Figure 1). Pathogens obtained in addition to *K. pneumoniae* from same patient were considered as co-infection. These included *P. aeruginosa* (*n* = 11), *Acinetobacter* spp. (*n* = 11), non-fermenting Gram-negative bacteria (NFGNB) (*n* = 4), *E. coli* (*n* = 5), *Klebsiella* spp. (*n* = 2), *Enterococcus* spp. (*n* = 5), *S. aureus* (*n* = 4), CoNS (*n* = 9), Group B *Streptococcus* (GBS) (*n* = 2), *Aeromonas* spp. (*n* = 1), and yeast (*n* = 12). There was no significant difference between the numbers in groups of strong, moderate, weak and negative biofilm producers.

### MBEC Analysis

**FIGURE 2:**
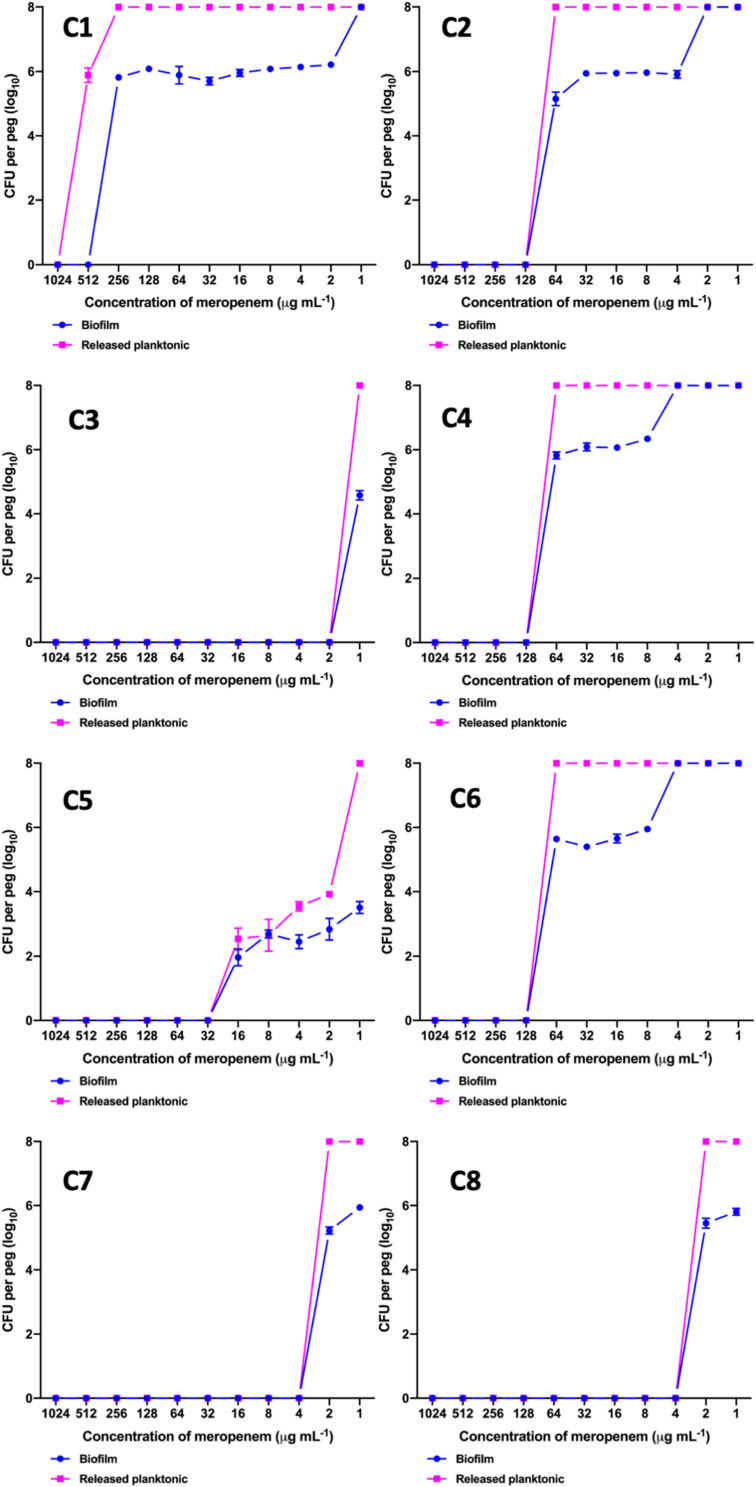
MBEC assay comparing the biofilm eradication efficiency of meropenem *in vitro* on *K. pneumoniae* with biofilm structure. *X*-axis indicates concentration of meropenem used for treatment, *Y*-axis indicates log_10_ CFU/ml of cells at respective concentrations. Data was visualized using GraphPad Prism v8.2.0. Biofilm – *K. pneumoniae* biofilm structure formed on pegs; Released planktonic – Cells released from the biofilm structure on pegs during meropenem treatment.

The MBEC of meropenem differed in comparison to MICs for the eight strong biofilm forming *K. pneumoniae* isolates tested (Table 2). The MBEC values for biofilms were higher than the MIC values estimated for planktonic cells. The quantitative log growth values (CFU/ml) of *K. pneumoniae* at various concentrations of meropenem were depicted in Figure 2. The values depict the actual representation of microbial population unaffected by meropenem in the biofilm structure. Isolates C1, C2, C4, and C6 had exhibited ≥128 μg/ml as MBEC values, whereas C3, C7, and C8 required ≤4 μg/ml for clearing the biofilms.

**TABLE 2:** MBEC values of meropenem toward biofilm forming clinical *K. pneumoniae*.

Isolate ID	*MIC by broth microdilution for planktonic cells*	*MBEC for biofilm*	*MBEC for planktonic released from biofilm*
C1	32 (R)	512 (R)	1024 (R)
C2	32 (R)	128 (R)	128 (R)
C3*	≤0.03 (S)	2 (I)	2 (I)
C4	16 (R)	128 (R)	128 (R)
C5*	≤0.03 (S)	32 (R)	32 (R)
C6*	≤0.03 (S)	128 (R)	128 (R)
C7	16 (R)	4 (R)	4 (R)
C8*	≤0.03 (S)	4 (R)	4 (R)

*Isolates with susceptible MIC but resistant to meropenem in biofilm structure (MBEC); R – resistant, I – intermediate, S – susceptible.

### Genome Analysis of *K. pneumoniae* and *K. quasipneumoniae*

A representative sample of six strong biofilm forming *K. pneumoniae* were sequenced to ∼60× coverage. WGS revealed three of these isolates to be *K. quasipneumoniae*. These were compared with global isolates obtained from NCBI for further analysis. Core genome analysis of 454 *K. pneumoniae* using Roary revealed 1129 core genes (in 99%), 1511 soft core (95–99%) and 3439 shell genes (15–95%), 36,072 cloud genes (<15%), total 42,151, whereas for 25 *K. quasipneumoniae*, 4,931 core genes, 1,394 shell genes, and 6,325 total genes were found. Total SNPS included for phylogenetic analysis were 1,80,537 for *K. pneumoniae* and 1,97,628 in *K. quasipneumoniae*.

### Association of Global Clones With Genes Responsible for Biofilm Mechanism and Carbapenem Resistance

**FIGURE 3:**
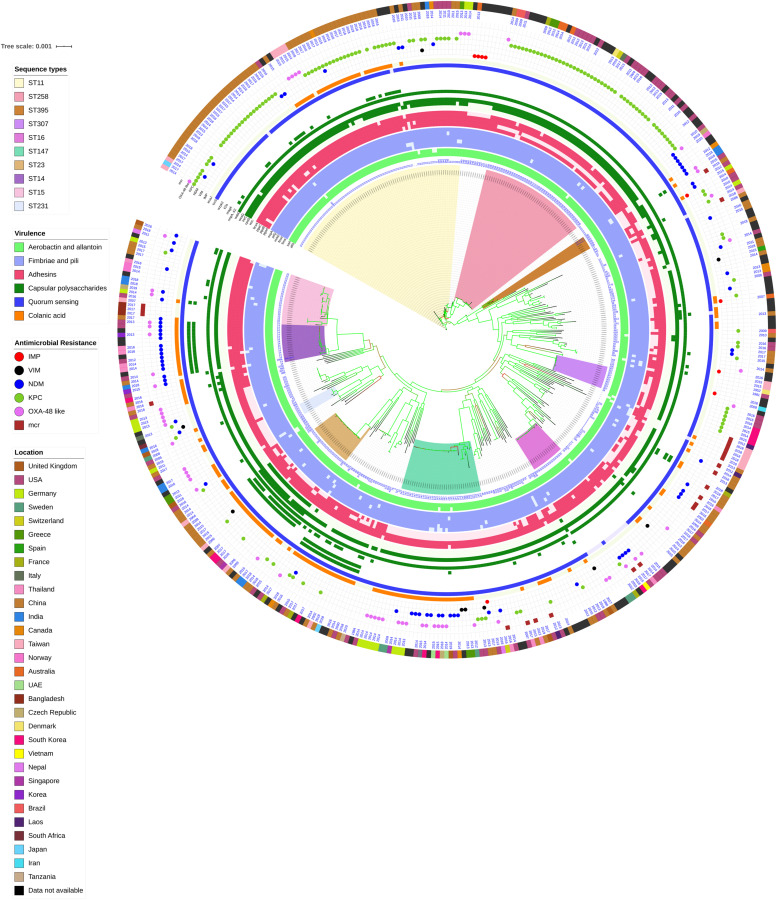
Core genome phylogeny of strong biofilm forming clinical *K. pneumoniae* in comparison to global genomes for identifying similarities in biofilm virulome and resistome. The color of branch leaves indicate the bootstrap values, green indicating high; red indicating low. STs were marked in color ranges; innermost circle represents biofilm virulome (*allS, iutA, fimA, fimH, mrkD, pilQ, ecpA, pgaABC, bcsA, wzc, cpsD, treC, wcaG, wabG, rmpA/A2, magA, k2a, wzyk2, luxS*, and *wcaJ*) followed by resistome (*bla*_IMP_, *bla*_VIM_, *bla*_NDM_, *bla*_KPC_, *bla*_OXA–__48_ like, and *mcr*) as shape plots. Location of each isolate has been given in the outermost ring as color strip.

**FIGURE 4:**
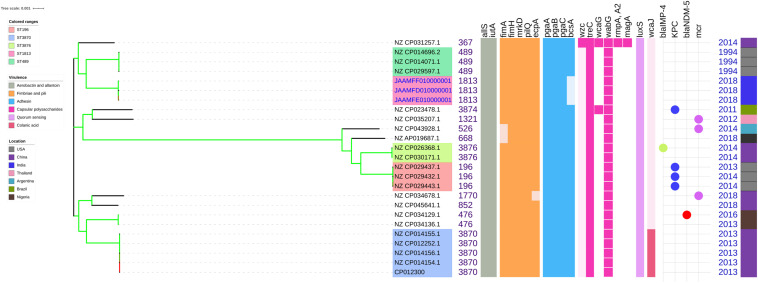
Comparison of *K. quasipneumoniae* clinical isolates to the global complete genomes indicating the biofilm virulome and resistome. The color of branch leaves indicate the bootstrap values, green indicating high; red indicating low. STs were marked in color ranges followed by biofilm virulome (*allS, iutA, fimA, fimH, mrkD, pilQ, ecpA, pgaABC, bcsA, wzc, cpsD, treC, wcaG, wabG, rmpA/A2, magA, k2a, wzyk2, luxS*, and *wcaJ*) and resistome (*bla*_IMP_, *bla*_VIM_, *bla*_NDM_, *bla*_KPC_, *bla*_OXA–__48_ like, and *mcr*). Color strips indicate the country of the isolate.

Among both *K. pneumoniae* and *K. quasipneumoniae*, genes responsible for biofilm formation, *allS* (aerobactin), *iutA* (allantoin), type 1 fimbriae (*fimA, fimH*), type III fimbriae (*mrkD*), pili (*pilQ, ecpA*), adhesins/polysaccharides (*pgaA, pgaB, pgaC*, and *bcsA*), CPS (*wzc, cpsD, treC, wcaG, wabG, rmpA/A2, magA, k2a*, and *wzyk2*), QS (*luxS*), colonic acid-mucoid (*wcaJ*) were screened and presented in Figures 3, 4, respectively. The AMR and biofilm gene profile of the six isolates were compared in Table 3. None of the isolates sequenced in this study harbored known mutations in the porin genes *ompK35*, *ompK36*, and *ompK37*.

**TABLE 3:** Comparison of AMR and biofilm gene profile between *K. pneumoniae* and *K. quasipneumoniae*.

Isolate ID	STs	ß-lactamases	Adhesin	Cohesion	CPS	QS	Colanic acid	Allantoin	aerobactin
C1	395	*bla*_CTX–M–__15_, *bla*_OXA–__1_, *bla*_SHV–__182_, ***bla*_NDM–__5_**	*fimA, fimH, mrkD, ecpA*	*pgaC*	*cpsD, wabG*	*luxS*	*wcaJ*	*allS*	*iutA*
C2	395	*bla*_CTX–M–__15_, *bla*_OXA–__1_, *bla*_TEM–__1__B_, *bla*_SHV–__11_, *bla*_SHV–__182_, ***bla*_NDM–__5_**, ***bla*_OXA–__232_**	*fimA, fimH, mrkD, ecpA*	*pgaC*	*cpsD, wabG*	*luxS*		*allS*	*iutA*
C4	2096	*bla*_CTX–M–__15_, *bla*_OXA–__1_, *bla*_DHA–__1_, *bla*_TEM–__1__A_, *bla*_SHV–__28_, *bla*_SHV–__106_, ***bla*_NDM–__5_**	*fimA, fimH, mrkD, pilQ*	*pgaB, pgaC*	*wabG, rmpA2*	*luxS*		*allS*	*iutA*
C3*	1813	*bla*_DHA–__1_, *bla*_OKP–B–__1_	*fimA, fimH, mrkD, pilQ, ecpA*	*pgaA, pgaB, pgaC*	*treC, wabG*	*luxS*		*allS*	*iutA*
C5*	1813	*bla*_DHA–__1_, *bla*_OKP–B–__1_	*fimA, fimH, mrkD, pilQ, ecpA*	*pgaA, pgaB, pgaC*	*treC, wabG*	*luxS*		*allS*	*iutA*
C6*	1813	*bla*_DHA–__1_, *bla*_OKP–B–__1_	*fimA, fimH, mrkD, pilQ, ecpA*	*pgaA, pgaB, pgaC*	*treC, wabG*	*luxS*		*allS*	*iutA*

*K. quasipneumoniae; susceptible to meropenem by MIC; STs – Sequence types.

The genes present among the *K. quasipneumoniae* isolates (C3, C5, and C6) justifies their susceptibility to carbapenems by lack of strong ß-lactamases, which is otherwise present in C1, C2, and C4 (*K. pneumoniae*). However, C3 was intermediate resistant; C5 and C6 were resistant to meropenem by MBEC assay. In addition, biofilm gene profile of *K. quasipneumoniae* were significantly different from *K. pneumoniae* for cohesion and CPS factors.

In *K. pneumoniae*, among 454 genomes (451 Genbank + 3 sequenced) the comparison of STs correlated well with the biofilm forming virulence genes, AMR genes and country of isolation. Among the sequenced isolates, two *K. pneumoniae* belonged to ST395, one *K. pneumoniae* belonged to ST2096 and three *K. quasipneumoniae* isolates belonged to ST1813. ST11 and ST258 (CC11) were the major clades harboring *bla*_KPC_ for carbapenem resistance. These clades were predominantly observed in the Americas, Europe and China. ST11 were predominantly seen in China, with only small numbers in United States, Europe and Taiwan. Isolates from Europe and Taiwan alone harbored *bla*_OXA–__48_ like carbapenemases instead of *bla*_KPC_. A fraction of isolates from China harbored *bla*_NDM_ either alone or in addition to *bla*_KPC_. In contrast, ST258 was a strict *bla*_KPC_ clone and did not harbor any other carbapenemases. ST258 was predominantly found in United States followed by fewer isolates in Europe and Australia.

ST11 harbored most of the genes required for the process of biofilm formation, *allS, iutA, fimA, fimH, mrkD, pilQ, ecpA, pgaA, pgaB, pgaC, bcsA, cpsD*, *treC*, and *wabG*. ST258 also carried the same set of genes for biofilm except *pgaB*, which was clearly missing in ST258. Both ST11 and ST258 did not harbor *wzc, wcaG, magA, k2a*, and *wzyk2* for CPS. *rmpA* and *rmpA2* were found in few of the ST11 isolates (*n* = 20) from China. Almost 50% of ST11 from China lacked *wcaJ* known for colanic acid production, while none of ST258 isolate carried *wcaJ*.

ST395 (CC395) was typically missing *pgaA* and *pgaB* genes for polysaccharide production. They were distributed in the United States (*n* = 2), India (*n* = 2), China (*n* = 1), and Germany (*n* = 1). One of the United States isolates carried *k2a* and *wzyk2* genes for K2 serotype. Two of the study isolates from India (C1 and C2) lacked *bcsA* and *treC* in addition to *pgaA* and *pgaB*. These isolates harbored only one gene for adhesin (*pgaC*), while carrying *cpsD*, *treC*, and *wabG* for CPS. Except two isolates from India (C1) and United States, none of ST395 carried *wcaJ*. Four out of six ST395 isolates harbored *bla*_NDM_, with one Indian isolate harboring both *bla*_NDM_ and *bla*_OXA–__232_. One of the two isolates lacking *bla*_NDM_ was found harboring *bla*_OXA–__48_ like. Interestingly, one isolate from China that completely lacked carbapenemases carried an *mcr* gene.

ST307 (CC307), which is again a *bla*_KPC_ predominant clone, was observed in the United States and South Korea. This clone lacked the *pgaB* gene for adhesin but carried *treC* and *wabG* for CPS. ST16 is an another reported MDR clone, observed in United States and Europe, with one isolate each in Thailand, Vietnam and South Korea. ST16 had a mixture of *bla*_NDM_ and *bla*_OXA–__48_ like carbapenemases. Interestingly, one isolate each from Thailand and Vietnam harbored *mcr* in addition to *bla*_NDM_. ST16 harbored only *treC* and *wabG* for CPS but harbored all other screened genes for type I and III fimbriae, adhesins, aerobactin and allantoin.

ST147 (CC147) is a reported *K. pneumoniae* global clone and was observed across all countries. However, based on the geographical occurrence, the carbapenemase genes carried differed. ST147 *K. pneumoniae* from Germany (*n* = 8), UAE (*n* = 2), Pakistan (*n* = 1), and Nepal (*n* = 1) harbored *bla*_OXA–__48_ like, while one isolate from Greece harbored *bla*_KPC_. *bla*_NDM_ was observed in ST147 from Singapore, Switzerland, United Kingdom and Canada. In addition, a combination of *bla*_NDM_ and *bla*_OXA–__48_ was observed in South Korea (*n* = 2) and Czech Republic (*n* = 1). ST147 from United States harbored both *bla*_NDM_ and *bla*_KPC_, while two isolates from China harbored each with *bla*_KPC_ + *bla*_VIM_ and *bla*_NDM_ + *bla*_IMP_. One ST147 isolate from Thailand carried an *mcr* gene for colistin resistance. ST147 from Sweden (*n* = 2) did not harbor any carbapenemases in 2009, later in 2012 an isolate found acquired *bla*_OXA–__48_ like. ST147 lacked *pgaA* and *pgaB* genes for adhesin, instead harbored *pgaC*, *bcsA*. For CPS ST147 carried only *treC* and *wabG*, while carrying *wcaJ* for colanic acid.

ST23 (CC23) was mostly found in China, with a few isolates identified in United States, South Korea and India. Few isolates from China and United States alone carried *bla*_KPC_, whereas one isolate from China and India carried *bla*_VIM_ and *bla*_OXA–__48_ like, respectively. ST23 harbored all screened genes for aerobactin, allantoin, fimbriae, pili, and adhesins except *pgaA*. For CPS, they exclusively carried *wzc* and *magA* gene in addition to *wabG* and *treC*, which was not seen in any related/unrelated STs. Also, *wcaG*, and *rmpA*/*A2* were seen majorly in ST23 with very few reports in other STs. Since these were the only ST harboring *wzc* genes for CPS, they were further analyzed for their efficiency in producing hyper-capsulation by carrying a 565 glycine-to-serine substitution in *wzc* genes. The analysis revealed that none of the isolates were mutated at 565 glycine-to-serine position, known for hyper-capsulation.

ST231 (CC43) and ST101 (CC11) are *bla*_OXA–__48_ carrying clones thought to be split from a single parent clade but still appear to converge in the core gene characteristics that are evident in their biofilm and AMR gene profiles. In this study population, ST231 was identified in India and the United States. Interestingly one isolate from ST101 carried *bla*_KPC_ from India. Both ST231 and ST101 lacked *pgaA* and *pgaB* genes for adhesins and carried only *treC* and *wabG* for CPS. Most of ST231 and ST101 lacked *wcaJ* for colanic acid. Similarly, ST14 (CC14) and ST15 (CC15) were diverged from a single parent clade found across most of the countries, where ST14 carried *bla*_NDM_ in almost all isolates (*n* = 17) in addition to *bla*_OXA–__48_ like in few (*n* = 5). Whereas, ST15 started to lose *bla*_NDM_ that only carried *bla*_NDM_ gene in 6 out of 23 isolates. ST14 differed from ST15 in their CPS gene profile and *wcaJ*. ST14 harbored *k2a* and *wzyk* responsible for K2 serotype along with *wcaJ* gene. In addition, the sequenced study isolate C4 belonging to ST2096 (variant of ST14) carried *rmpA* and *rmpA2*, while lacking *treC*. Among ST15, only one isolate from United States carried *k2a*, *wzyk2*, and *wcaJ* while others lacked these genes. Five out of 23 isolates harbored *rmpA* and *rmpA2*.

**FIGURE 5:**
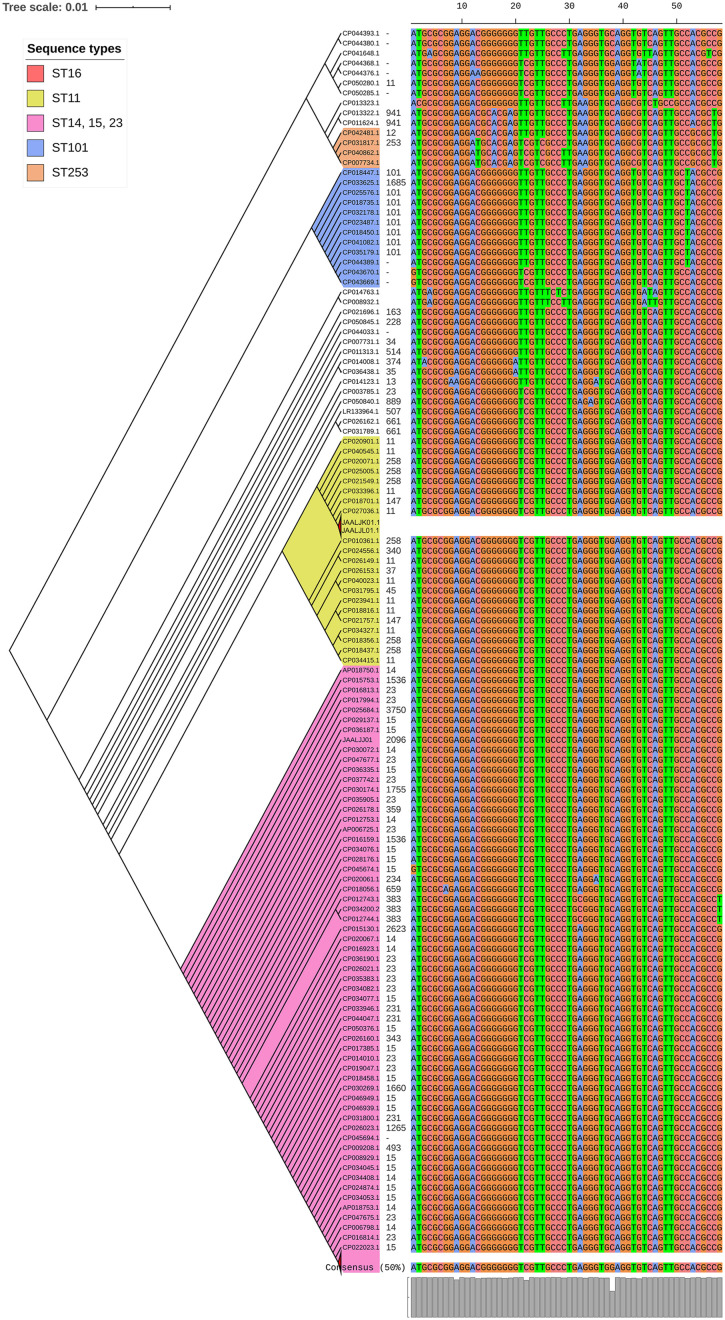
The phylogeny of *mrkD* was based on the maximum-likelihood tree with 59 SNPs from 451 *K. pneumoniae*. Isolates with similar SNP pattern were clustered together for better representation. Six major variants were observed from the multi-alignment of SNPs. The STs did not exactly correlate with the variants of *mrkD*, whereas multiples STs share same *mrkD* variants.

**FIGURE 6:**
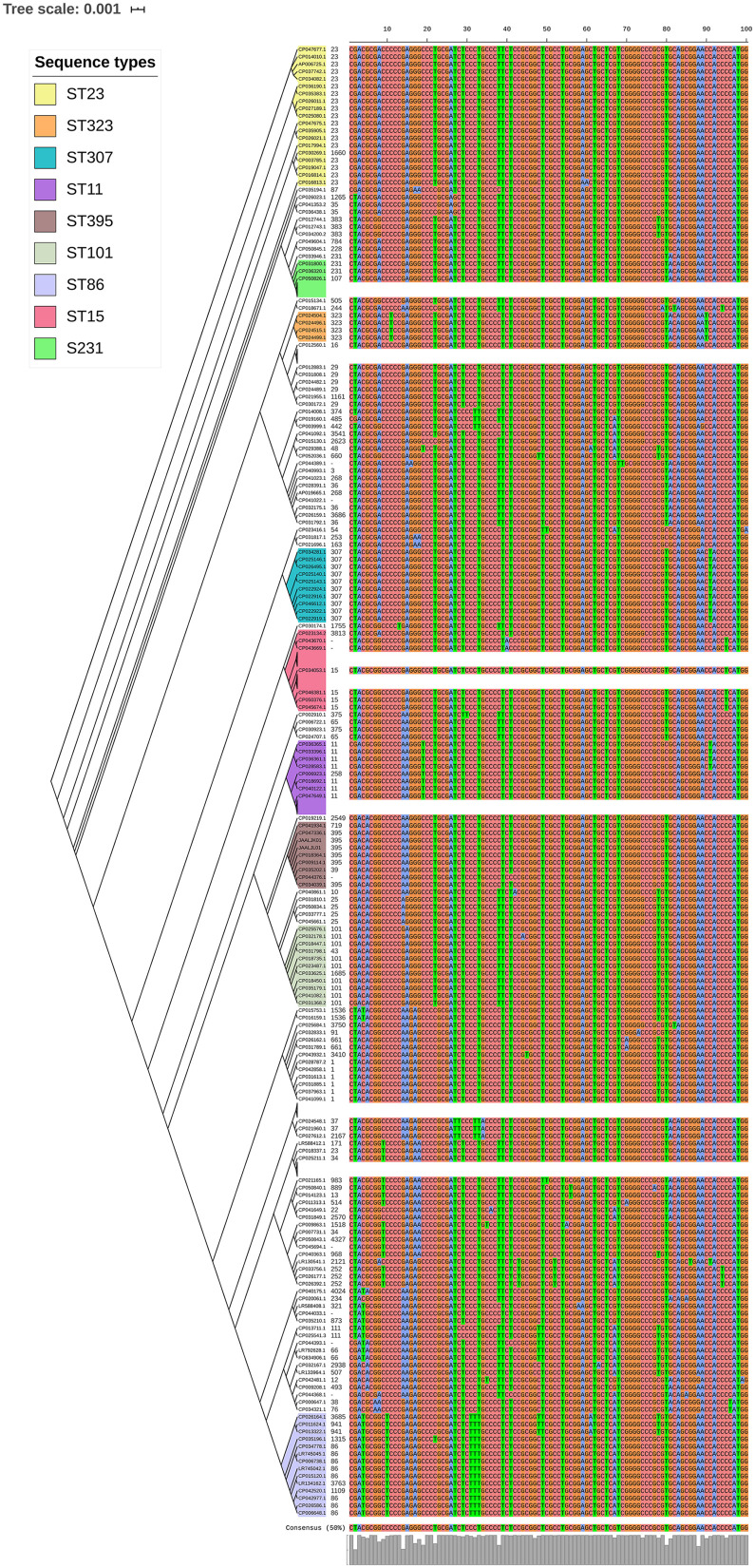
*treC* phylogeny was based on the maximum-likelihood tree with 135 SNPs from 451 *K. pneumoniae*. Isolates with similar SNP pattern were clustered together for better representation. The STs correlate with the *treC* variants observed. Sequenced isolates C1, C2, and C4 did not harbor *treC* gene.

*luxS* responsible for QS in *K. pneumoniae* was present in almost all isolates irrespective of STs and countries. *mrkD*, and *treC* were further analyzed for differences in mutation. *mrkD* SNP analysis revealed six major *mrkD* variants among the 451 *K. pneumoniae* with 59 SNPS (Figure 5). In contrast, most of the *treC* were mutated and the variants observed were diverse with 135 SNPs (Figure 6). However, observed variants of *treC* were strongly associated with the specific STs unlike *mrkD*.

Unlike *K. pneumoniae*, in *K. quasipneumoniae* each ST was observed to be specific to each country. ST1813 was found in India (C3, C5, and C6), ST489 and ST196 were found in United States, whereas ST3870 and ST3876 were found in China. All isolates harbored *allS, iutA, fimH, mrkD, pilQ, ecpA, pgaA, pgaB, pgaC, treC, wabG*, and *luxS*. ST526 and ST668 lacked the type I fimbriae gene *fimA*, wheres ST1818 from India lacked *bcsA* gene responsible for adhesin. Except one, none of the isolates harbored *wzc* for CPS but harbored *treC* and *wabG* instead. QS2 family gene *luxS* was found in all isolates. Except ST3870, none of the isolates harbored *wcaJ* gene.

**FIGURE 7:**
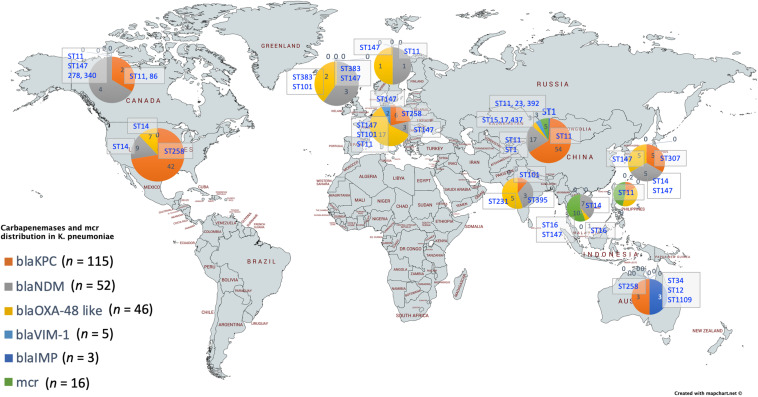
Country-wise distribution of carbapenemases and *mcr* genes in *K. pneumoniae*. Map outline was created using mapchart.net (https://mapchart.net/).

**FIGURE 8:**
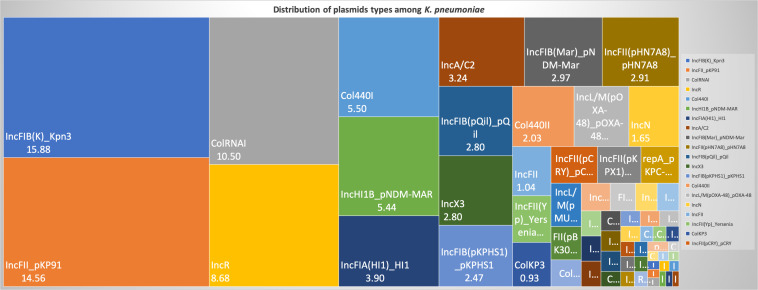
Distribution of plasmid types (percentage) among 454 *K. pneumoniae*. A total of 1286 plasmids were harbored in 454 isolates with 1819 rep types. Plasmids harboring single replicon type were 791, two replicons – 457, three replicons – 34, and four replicons were three plasmids.

For a better understanding of AMR genes across the globe, country-wise distribution of carbapenemases and *mcr* genes along with STs identified in the 454 *K. pneumoniae* genomes analyzed in this study was depicted in Figure 7. *bla*_KPC_ was the dominant carbapenemase in United States and China, whereas *bla*_NDM_ was more or equal to *bla*_OXA–__48_ like genes in United Kingdom, Norway, Sweden, Japan and Korea. In contrast, among India and Europe *bla*_OXA–__48_ like was common. In Australia, both *bla*_IMP_ and *bla*_KPC_ were observed. Interestingly, *mcr* genes were observed in China, Taiwan, Thailand and Vietnam. Distribution of plasmids among *K. pneumoniae* are depicted in Figure 8. A total of 1286 plasmids were harbored in 454 isolates with 1819 replicon types with IncFIB(K) and IncFII being the most common. All six sequenced isolates commonly harbored IncFIB(K)_Kpn3 plasmids in addition to other replicon types.

Moreover, the association of genetic biofilm factors and AMR genes were analyzed using scoary (Table 4). Genes observed significant (*p* < 0.05) with Benjamini-Hochberg’s *p*-value were only considered. *bla*_IMP_ and *blaVIM* did not associate with any of the screened biofilm genetic factors, whereas *bla*_OXA–__48_ like, *bla*_NDM_ and *bla*_KPC_ had significant associations with biofilm genes co-carried with them.

**TABLE 4:** Association of biofilm genetic virulence factors with antimicrobial resistance genes for carbapenem resistance in 454 *K. pneumoniae* isolates as analyzed using scoary.

		*p*-values
*bla*_OXA–48_ like	*mrkD*^+^	0.01*
	*BcsA*	0.08
	*WcaJ*	0.05*
	*pgaA*^+^	0.05*
*bla*_NDM_	*mrkD*^+^	0.03*
	*fimA*	0.01*
	*pgaC*	0.09
	*wcaJ*	0.03*
	*iutA*	0.04*
*bla*_KPC_	*mrkD*	0.03*
	*fimA*	0.06
	*fimH*	0.03*
	*treC*	0.09
*mcr*	*bcsA*	0.02*
	*bcsB*	0.002*
	*iutA*	0.05*

^+^hypothetical proteins were significantly co-carried with these biofilm genetic factors. *p < 0.05; *p < 0.01.

## Discussion

The biofilm-forming capacity of clinical *K. pneumoniae* isolates had a significant association with the outcome in respective patients. Strong biofilm formation significantly reduced the number of days alive for the patient to 3.33 days from the poor/negative biofilm producing isolates with 11.33 days. This clearly demonstrates the effect of biofilm formation as an association for increasing/accelerating the mortality. This is of particular concern, as *K. pneumoniae* can survive for prolonged periods in the hospital environment and grow attached to inanimate surfaces like medical devices; catheters and ventilators (Murphy and Clegg, 2012).

In such situations, polymicrobial infections might further complicate the therapy. Monomicrobial or polymicrobial isolates were identified in addition to *K. pneumoniae* among 45 out of 72 patients. This includes, *P. aeruginosa*, *Acinetobacter* spp., *Enterococcus* spp., *S. aureus*, and *Candida* spp., *P. aeruginosa* and *A. baumannii* were the most commonly co-infected bacterial species followed by equal number of yeasts in these patients. This could be attributed to the known persistence of these pathogens in a hospital environment and their reported ability for biofilm formation. Polymicrobial infections are known to play an inevitable role in managing biofilm structure and causing persistent infection, thereby challenging appropriate antimicrobial therapy.

The biofilm structure has been known to fuel/protect the pathogen even in stressful conditions *in vivo*, thereby resulting in clinical failure irrespective of antimicrobial susceptibility. This was clearly reflected in the 35% (*n* = 5) of clinical failure among the carbapenem-susceptible infections (*n* = 14); three of the five clinical failures were strong or moderate biofilm formers. Of the remaining two, one patient had diabetes mellitus as a comorbid condition while the other did not show any co-morbid condition. However, more numbers are required to statistically confirm this association. The clinical failure might be due to a hypervirulent condition that needs further analysis for confirmation. Treatment failure was observed in a proportion of patients who received carbapenem monotherapy due to development of resistance, particularly in those infected with biofilm forming strains. This shows that biofilm producing strains develops resistance rapidly upon antibiotic exposure and thus lead to addition or change of antimicrobial agent. For CR *K. pneumoniae*, colistin in combination with meropenem was prescribed. Further, for carbapenem-colistin resistant *K. pneumoniae*, aminoglycosides or tigecycline was added in combination for therapy.

The MBEC assay also confirms the findings, as C3, C5, C6 and C8 strains showed an increase from MICs of ≤0.03 μg/ml (planktonic cells) to 2, 32, 128, and 4 μg/ml, respectively for biofilms. These susceptibility results of carbapenem on the fully-grown biofilm structure were consistent with the previous reports for gentamicin, cefotaxime and ciprofloxacin, amikacin and piperacillin in *K. pneumoniae* (Bellifa et al., 2013; Singla et al., 2013). These were further validated by their AMR gene composition, as C3, C5, and C6 (susceptible by MIC) harbored only *bla*_DHA_ and *bla*_OKP–B_, whereas C1, C2 and C4 harbored strong carbapenemases such as *bla*_NDM–__5_ and *bla*_OXA–__232_. However, C3 was intermediate resistant; C5 and C6 were resistant to meropenem by MBEC assay, exhibiting the complexity of antimicrobial action on biofilm structures. Remarkably, MBEC assays further revealed that the planktonic cells released from mature biofilm structure exhibited the same MICs as the biofilm cells, which might possibly be a mechanism for establishment of mobile resistant population *in vivo* causing clinical failure. In contrast, as the MBEC levels are far greater than the MICs, MICs cannot be extrapolated for treatment of biofilm-mediated infections in the early stages. This signifies the importance of an MBEC screening to determine the appropriate antimicrobial dosing strength, which might require a combination therapy or an increased dose of monotherapy.

Several scientific investigations have hypothesized various physical mechanisms involved in promoting AMR due to biofilm formation. One of the commonly proposed methods has been the ability of biofilm matrix to prevent efficient diffusion of antibiotics, leading to significantly decreased exposure of bacteria in biofilms, in addition to development of resistance due to altered gene expression of antibiotic tolerance genes, and horizontal transfer of AMR genes between cells in a biofilm environment (Lewis, 2008; Lazar and Chifiriuc, 2010). In *K. pneumoniae*, it was reported that ampicillin was steadily degraded as it diffused through biofilm matrix. Ciprofloxacin could penetrate the biofilm but could not kill the bacterial cells as they become tolerant at nutrient limited conditions (Anderl et al., 2000, 2003). In addition, antibiotics piperacillin, piperacillin-tazobactam, cefoperazone, ceftazidime, cefepime, meropenem, ciprofloxacin, netilmicin and amikacin were proven to exhibit reduced activity against adherent bacteria when compared to the planktonic counterparts (Cernohorská and Votava, 2004).

The genetic mechanisms known to be involved for a successful biofilm formation in *K. pneumoniae* include factors for adhesion (fimbriae and pili) (Alcántar-Curiel et al., 2013), cohesion (adhesins, polysaccharides), CPS (Wu et al., 2011), QS (Chen et al., 2020), and loss of mucoidal nature (colanic acid) (Pal et al., 2019). Here, genes responsible for each of these factors were analyzed for the study isolates in comparison with global clones. Though a few reports suggest recombination between *K. variicola* and *K. quasipneumoniae* (Long et al., 2017), and between *K. quasipneumoniae* and *K. pneumoniae* (Holt et al., 2015), most of the studies report that homologous recombination does not occur between strains in the different clades (Brisse et al., 2014). Similarly, the phylogenetic analysis in the present study revealed significant differences between *K. pneumoniae* and *K. quasipneumoniae* and hence they were studied separately for further analysis.

Fimbriae and pili structures are known for their ability to support motility as well as attachment to a biotic or an abiotic surface, essential in the initial stages of biofilm formation. Firstly, it was considered that type I fimbriae are not as important as type III fimbriae for biofilm formation. This was due to exclusive adhesive property by MrkD protein at the tip of the fimbriae, which aids in pathogenesis by better attachment of *K. pneumoniae*, (i) to the basolateral surfaces *in vivo* such as urinary tract or bronchial epithelia (Murphy and Clegg, 2012); (ii) to damaged mucosal surfaces caused due to insertion of indwelling devices like catheters, as well as adherence to the surfaces of these devices which would be coated with host-derived conjugates (Tarkkanen et al., 1990; Miettinen et al., 1993). However, recent investigations have indicated that either type I or type III fimbriae may play a role in biofilm formation (Stahlhut et al., 2012). Accordingly, in the present study all six sequenced isolates harbored genes responsible for both type I (*fimA* and *fim*H) and type III (*mkr*D) fimbriae. Also, these genes were present irrespective of STs across all global clones.

Several allelic variants of *mrkD* have been identified in different *Klebsiella* isolates. These alleles are known to be associated with different binding specificities to matrix material such as collagen (Huang et al., 2006). Interestingly, isolates belonging to different STs shared the same *mrkD* variant. ST11 and ST258, in addition to belonging in the same CC (CC11), shared the same *mrkD* variant. Moreover, the sequenced isolates JAALJK0.1 and JAALJ01.1 from a different ST (ST395) also belonged to the same clade of *mrkD*. The low number of variants observed in *mrkD* of 454 *K. pneumoniae* exhibit them as a stable genetic factor for biofilm mechanism across all STs.

The *E. coli* common pilus (*ecp*) and *pilQ* are known to be important in regulation of pili function in *K. pneumoniae*. In the present study, *ecpA* and *pilQ* were observed in almost all isolates of *K. pneumoniae* and *K. quasipneumoniae*. Similarly, Alcántar-Curiel et al. (2013) reported that 96% of the *K. pneumoniae* strains contained *ecpA* with a 94% phenotypic correlation of ECP production crucial to form an adhesive structure on cultured epithelial cells. Among *K. pneumoniae* sequenced in this study, one ST2096 (CC14) was negative for *ecpA*, while *pilQ* was absent only in two of the *K. pneumoniae* belonging to ST395. In *K. quasipneumoniae*, all three isolates harbored both *ecpA* and *pilQ* genes.

Although genes responsible for adhering structure were shown to be present, the actual secretory polysaccharides and other adhesins play a critical role in physical attachment that enhances biofilm formation. *pgaABCD* and *bcsA* were highly reported adhesins in *K. pneumoniae* (Chen K. M. et al., 2014; Wang et al., 2016). The adhesin gene profile correlated with the particular STs. Accordingly, ST11, ST15, ST14, and ST16 carried all four genes; ST258 and ST307 carried *pgaA*, *pgaC*, and *bcsA*; ST23 carried *pgaB*, *pgaC*, and *bcsA*; while ST101, ST147, ST231, and ST395 carried only *pgaC* and *bcsA*. Chen K. M. et al. (2014) previously reported that the loss of *pgaC* affected the production of poly-β-linked N-acetylglucosamine which in turn had inhibitory effects on *in vitro* biofilm formation. Among the six sequenced isolates, none of them harbored *bcsA*. *K. quasipneumoniae* harbored all three *pga* gens, while in *K. pneumoniae* two isolates harbored *pgaC* and one harbored *pgaB* and *pgaC* genes.

Lipopolysaccharides are known to be involved in the initial adhesion on abiotic surfaces and capsule plays a critical role in construction of mature biofilm architecture (Balestrino et al., 2008). *wzc*, *cpsD*, and *treC* have been reported to be key regulators in the CPS production (Wu et al., 2011). Since ST23 were the only isolates found to harbor *wzc* genes in the study population, they were further analyzed for their efficiency in hyper-capsulation due to a 565 glycine-to-serine substitution in *wzc* genes. None of the ST23 isolates carried Gly-565Ser substitution, known for hyper-capsulation. A recent study by Ernst et al. (2020) revealed that disruption/absence of *wzc* might cause the hyper-capsulation required for biofilm formation irrespective of *rmpA/A2*. Accordingly, ST23 had genes exclusively for hypervirulence characteristics such as *wcaG, magA*, and *rmpA*/*A2*, although they might not be good biofilm producers.

*treC* was found to be crucial for capsule production and biofilm formation via trehalose utilization/CPS modulation (Wu et al., 2011). *treC* was identified in almost all study isolates of *K. pneumoniae* and *K. quasipneumoniae*. Though, there was a correlation between the STs and SNPs within *treC* gene; the variants observed were diverse. Moreover, variants of *mrkD*, *wzc* and *treC* genes in these isolates requires further gene knock-out studies to analyze the effect of these variants among clinical *K. pneumoniae*.

Furthermore, factors well known for hypervirulence, such as *allS*, *iutA*, *rmpA*/*A2*, *magA*, *K2A*, *wabG*, and *wcaG* were also reported for their role in biofilm formation and *magA*, *K2a*, *rmpA*/*A2*, *wabG*, and *wcaG* were shown to regulate CPS synthesis (Zheng et al., 2018; Hasani et al., 2020). In fact, *wcaG* was proven to be an independent risk factor for biofilm formation and highly associated with ST23 (Zheng et al., 2018). In line with this, these factors were also identified in isolates analyzed in this study. *wcaG* and *magA* were exclusive to ST23, whereas *rmpA* and *rmpA2* were seen majorly in ST23, in addition to C4 (ST2096) and other few STs. Almost all *rmpA/A2* observed were plasmid mediated.

QS is a well-established mechanism in the process of biofilm formation, where the population is regulated on sensing the cell-cell contact (Chen et al., 2020). Type II QS system reported in *K. pneumoniae* showed the role of *luxS* in autoinducer AI-2 synthesis (Balestrino et al., 2005; Zhu et al., 2011). It was also noted that changes in biofilm architecture were observed in the *luxS* mutant *K. pneumoniae* with less surface coverage and reduced macrocolony formation (Chen et al., 2020). *luxS* was present in almost all isolates in the study irrespective of STs.

A recent study by Pal et al. (2019) reported that loss or disrupted *wcaJ* (glycosyltransferase for colonic acid production) in *K. pneumoniae* made the isolates less mucoidal and higher in biofilm forming efficiency leading to reduced susceptibility toward both polymyxins and macrophages (less immunogenic). Except ST3870, none of the *K. quasipneumoniae* harbored *wcaJ* gene. While in *K. pneumoniae*, *wcaJ* was restricted to ST14, ST23, ST147, and part of ST11. Interestingly, ST15, ST16, ST307, and ST258, reported global high-risk clones, were *wcaJ* negative indicating the high potential of biofilm forming capacity in these STs. Sequenced isolates did not harbor *wcaJ* except C1 (ST395).

The common ST observed among the three *K. quasipneumoniae* (ST1813) suggested the common structural genome backbone which is reflected by carrying of same biofilm and ß-lactamase genes. However, the ß-lactamase genes observed in *K. pneumoniae* were different due to the diverse plasmid carrying capacities of *K. pneumoniae* irrespective of the same sequence type. Some of the previous studies report association of phenotypic biofilm forming efficiency with the AMR genes in *K. pneumoniae* (Vuotto et al., 2014). In clinical *K. pneumoniae*, it was reported that 44.7% were biofilm formers and 45.3% were ESBLs producers (Yang and Zhang, 2008). However, there was a lacuna of genetic factors being compared for biofilm mechanism with such AMR genes. Results observed in this study clearly exhibit the strong association of genes responsible for biofilm formation and AMR. Though, *bla*_IMP_ and *bla*_VIM_ did not associate with any of the screened biofilm genetic factors, it was evident that *bla*_OXA–__48_ like, *bla*_NDM_ and *bla*_KPC_ had significant (*p* < 0.05) associations with biofilm genes, especially *mrkD* with all three genes. In addition, gene encoding hypothetical proteins with significant (*p* < 0.05) associations alongside *mrkD* and *pgaA* were identified. Gene knockout studies on these genes encoding hypothetical proteins will reveal their role in regulating biofilm formation. Further, expression analysis is need for better comparison of strong, moderate, and non-biofilm producing strains.

Limitations of the study include, that the study retrospectively used isolates obtained from blood or endotracheal aspirate for biofilm screening. Also, the study included only complete genomes from NCBI to generate a high-quality phylogeny output so as to avoid bias in the core vs accessory genes being characterized which occurred with the shot-gun genome sequences (data not shown). The small number of complete genomes available in few of the STs included in the analysis significantly limited the ability to arrive at definitive conclusions on certain gene correlations.

## Conclusion

A single genetic factor is not always adequate, rather it requires a set of genetic factors to facilitate the complete formation of biofilm. Accordingly, this study discussed various genetic factors involved in biofilm formation of *K. pneumoniae* and *K. quasipneumoniae*, which cluster to provide a combined effect called biofilm. These results highlight the importance of biofilm testing, especially for nosocomial infections that are difficult to clear *in vivo*. Accordingly, genes *mrkD*, *wcaJ*, *pgaA*, and *pgaC* in addition to *fimA*, *fimH*, and *treC* could be potential screening RT-PCR markers for rapid diagnostics of biofilms in *K. pneumoniae*. These infections require additional treatment that might effectively help in improving patient outcome. Further, information on the clonal spread of biofilm forming *K. pneumoniae* across the globe will help to understand the dynamics of biofilm infections thereby paving way for effective management of nosocomial infections.

## Data Availability Statement

The datasets presented in this study can be found in online repositories. The names of the repository/repositories and accession number(s) can be found in the article/Supplementary Material.

## Author Contributions

ND, BV, PM, and EK conceptualized the study. ND, DM, and YU performed the experimental work and collected the data. The data were analyzed by ND and DPMS. ND performed all bioinformatic analyses. ND and DPMS wrote the manuscript, reviewed by BV, EK, PM, and HT. BV, PM, and EK supervised the project, experimental design, the data collection and analysis, and manuscript preparation. All authors contributed to the article and approved the submitted version.

## Conflict of Interest

The authors declare that the research was conducted in the absence of any commercial or financial relationships that could be construed as a potential conflict of interest.

## Footnotes

1 https://www.ncbi.nlm.nih.gov/genome/annotation_prok/

2 http://www.cge.cbs.dtu.dk/services

3 https://github.com/tseemann/snippy

4 https://github.com/tseemann/mlst

5 https://mapchart.net/
