# Supplementary material for: The Influence of Biofilms on Carbapenem Susceptibility and Patient Outcome in Device Associated K. pneumoniae Infections: Insights Into Phenotype vs Genome-Wide Analysis and Correlation
Source: Front Microbiol. 2020 Dec 14;11:591679. doi: 10.3389/fmicb.2020.591679 (PMC7767932; doi:10.3389/fmicb.2020.591679)
Supplement: Supplementary file 1 [file Table_1.DOCX]

**Supplementary Table 1:** Clinical outcome, co-morbidity, biofilm screening and antimicrobial resistance pattern of the 72 *K. pneumoniae* study isolates

| **Sample ID** | **Age** | **Sex** | **Outcome** | **Co-Morbidity** | **Days between infection and outcome** | **Sample type** | **Biofilm screening**  **assay** | **Antimicrobial resistance profile** |
| --- | --- | --- | --- | --- | --- | --- | --- | --- |
| VB1 | 17 | M | Expired | Nil | 4 | Blood | Weak | FOX-CFP/SUL-CHL-FEP-CPD-FEP-CTX-CAZ-ERT-GEN-AMK-HLG-IMI-LEV-LIN-MERO-NET-PTZ |
| VB2 | 68 | M | Expired | Diabetes mellitus | 2 | Blood | Strong | FOX-CFP/SUL-CHL-CIP-CPD-FEP-CTX-CAZ-ERT-GEN-AMK-HLG-IMI-LEV-LIN-MERO-MIN-NET-PTZ-TET |
| VB3 | 56 | F | Expired | kidney injury | 1 | Blood | Strong | FOX-CEF/SUL-CIP-CPD-FEP-CTX-CAZ-ERT-GEN-AMK-MERO-MIN-NET-PTZ-TET-TIGI |
| VB4 | 76 | M | Expired | Nil | 4 | Blood | negative | FOX-CEF/SUL-CHL-CIP-CPD-FEP-CTX-CAZ-ERT-GEN-MERO-MIN-PTZ-TET |
| VB5 | 32 | F | Discharged | liver injury | 19 | Blood | negative | FOX-CEF/SUL-CHL-CIP-CPD-FEP-CTX-CAZ-ERT-GEN-AMK-MERO-MIN-NET-PTZ-TET |
| VB6 | 17 | M | Expired | Nil | 1 | Blood | Strong | FOX-CEF/SUL-CHL-CIP-CPD-FEP-ERT-GEN-AMK-MERO-NET-PTZ-TET |
| VB7 | 61 | M | Expired | Nil | 11 | Blood | Moderate | FOX-CEF/SUL-CHL-CIP-CPD-CTX-CAZ-ERT-GEN-AMK-MERO-MIN-NET-PTZ-TET |
| VB8 | 36 | F | Expired | Nil | 6 | Blood | negative | FOX-CEF/SUL-CHL-CIP-CPD-FEP-CTX-CAZ-ERT-GEN-AMK-MERO-MIN-NET-PTZ-TET |
| VB9 | 19 | F | Discharged | Trachea NG tube removal | 21 | Blood | Moderate | CEF/SUL-CHL-CIP-CPD-FEP-CTX-CAZ-ERT-GEN-AMK-MERO-MIN-NET-PTZ-TET-TIGI |
| VB10 | 53 | F | Expired | Nil | 5 | Blood | negative | FOX-CEF/SUL-CHL-CIP-CPD-FEP-CTX-CAZ-ERT-GEN-AMK-MERO-NET-PTZ-TET |
| VB11 | 36 | F | Expired | Nil | 3 | Blood | negative | FOX-CEF/SUL-CHL-CIP-CPD-FEP-CTX-CAZ-ERT-GEN-AMK-MERO-NET-PTZ-TET |
| VB12  (C3) | 22 | F | Expired | Nil | 1 | Blood | Strong | CPD-CTX-CAZ-IMI |
| VB13 | 19 | M | Discharged | Kidney transplant | 39 | Blood | negative | FOX-CEF/SUL-CHL-CIP-CPD-FEP-CTX-CAZ-ERT-GEN-AMK-MERO-MIN-NET-PTZ-TET |
| VB14 | 18 | F | Expired | Nil | 2 | Blood | Moderate | FOX-CFP/SUL-CHL-CIP-CPD-FEP-CTX-CAZ-GEN-AMK-IMI-MERO-MIN-NET-PTZ-TET |
| VB15 | 38 | M | Discharged | Hypertension | 17 | Blood | Strong | All susceptible |
| VB16 | 50 | F | Discharged | Nil | 14 | Blood | Weak | FOX-CEF/SUL-CIP-CPD-FEP-CTX-CAZ-ERT-GEN-AMK-MERO-NET-PTZ-TET |
| VB17 | 55 | M | Expired | Nil | 1 | Blood | Moderate | FOX-CEF/SUL-CHL-CIP-CPD-FEP-CTX-CAZ-ERT-GEN-AMK-MERO-MIN-NET-PTZ-TET |
| VB18 | 45 | F | Discharged | Nil | 4 | Blood | negative | FOX-CEF/SUL-CHL-CIP-CPD-FEP-CTX-CAZ-ERT-GEN-AMK-MERO-NET-PTZ-TET-TIGI |
| VB19 | 47 | M | Discharged | Diabetes mellitus | 8 | Blood | Moderate | FOX-CFP/SUL-CHL-CIP-CPD-FEP-CTX-CAZ-GEN-AMK-IMI-MERO-MIN-NET-PTZ-TET-TIGI |
| VB20 | 55 | M | Discharged | Diabetes mellitus | 15 | Blood | Moderate | FOX-CFP/SUL-CHL-CIP-CPD-FEP-CTX-CAZ-GEN-AMK-IMI-MERO-NET-PTZ-TET |
| VB21 | 59 | M | Expired | Type 2 Diabetes mellitus | 5 | Blood | negative | FOX-CEF/SUL-CHL-CIP-CPD-FEP-CTX-CAZ-ERT-GEN-AMK-MERO-MIN-NET-PTZ-TET |
| VB22 | 63 | M | Expired | Nil | 1 | Blood | Weak | FOX-CEF/SUL-CHL-CIP-CPD-FEP-CTX-CAZ-ERT-GEN-AMK-MERO-MIN-NET-PTZ-TET |
| VB23 | 55 | M | Discharged | Nil | 25 | Blood | Strong | FOX-CEF/SUL-CHL-CIP-CPD-FEP-CTX-CAZ-ERT-GEN-AMK-MERO-MIN-NET-PTZ-TET |
| VB24  (C4) | 30 | M | Expired | Nil | 3 | Blood | Strong | FOX-CFP/SUL-CIP-CPD-FEP-CTX-CAZ-GEN-AMK-IMI-MERO-MIN-NET-PTZ-TET |
| VB25 | 45 | M | Expired | Nil | 9 | Blood | Weak | FOX-CEF/SUL-CHL-CIP-CPD-CTX-CAZ-ERT-GEN-MERO-MIN-PTZ-TET |
| VB26 | 25 | F | Expired | Nil | 25 | Blood | negative | FOX-CIP-CPD-FEP-CTX-CAZ |
| VB27 | 62 | F | Expired | Nil | 7 | Blood | Moderate | FOX-CFP/SUL-CHL-CIP-CPD-FEP-CTX-CAZ-ERT-AMK-MERO-MIN-NET-PTZ-TET |
| VB28 | 55 | F | Expired | Diabetes mellitus | 3 | Blood | Moderate | FOX-CFP/SUL-CHL-CIP-CPD-FEP-CTX-CAZ-ERT-GEN-AMK-MERO-MIN-NET-PTZ-TET-TIGI |
| VB29 | 45 | F | Expired | Nil | 2 | Blood | Weak | FOX-CFP/SUL-CHL-CIP-CPD-FEP-CTX-CAZ-ERT-GEN-AMK-MERO-MIN-NET-PTZ-TET-TIGI |
| VB30  (C5) | 56 | F | Discharged | Stem cell transplantation | 1 | Blood | Strong | FOX-CFP/SUL-CHL-CIP-CPD-FEP-CTX-CAZ-ERT-GEN-MERO-MIN-PTZ-TET-TIGI |
| VB31  (C6) | 57 | M | Discharged | Diabetes mellitus | 9 | Blood | Strong | FOX-CHL-CIP-CPD-FEP-CTX-CAZ-GEN-AMK-IMI-MERO-NET-PTZ-TET-TIGI |
| VB32 | 30 | M | Expired | Nil | 57 | Blood | Strong | CPD-CTX-CAZ |
| VB33 | 46 | M | Expired | Stem cell transplantation | 5 | Blood | Moderate | FOX-CEF/SUL-CHL-CIP-CPD-FEP-CTX-CAZ-ERT-GEN-AMK-MERO-MIN-NET-PTZ-TET |
| VB34 | 50 | M | Discharged | Diabetic | 6 | Blood | Moderate | FOX-CEF/SUL-CHL-CIP-CPD-FEP-CTX-CAZ-ERT-AMK-MERO-NET-PTZ-TET |
| VB35 | 37 | M | Discharged | Nil | 3 | Blood | Moderate | All susceptible |
| VB36 | 74 | F | Discharged | Nil | 1 | Blood | Strong | FOX-CEF/SUL-CIP-CPD-FEP-CTX-CAZ-ERT-GEN-AMK-MERO-MIN-NET-PTZ |
| VB37 | 30 | F | Discharged | vitamin B12 | 83 | Blood | Strong | FOX-CEF/SUL-CHL-CIP-CPD-FEP-CTX-CAZ-ERT-GEN-AMK-MERO-NET-PTZ-TET |
| VB38 | 54 | M | Expired | Diabetes mellitus | 9 | Blood | Weak | TET |
| VB39  (C1) | 59 | M | Expired | Diabetes mellitus | 6 | Blood | Strong | FOX-CFP/SUL-CHL-CIP-CPD-FEP-CTX-CAZ-ERT-GEN-AMK-MERO-NET-PTZ-TET |
| VB40  (C2) | 54 | M | Expired | Nil | 9 | Blood | Strong | FOX-CFP/SUL-CHL-CIP-CPD-FEP-CTX-CAZ-ERT-GEN-AMK-MERO-MIN-NET-PTZ-TET |
| VB41 | 38 | M | Discharged | Hypertension | 11 | Blood | Strong | FOX-CEF/SUL-CHL-CIP-CPD-FEP-CTX-CAZ-ERT-GEN-AMK-MERO-MIN-NET-PTZ-TET |
| VB42 | 45 | F | Expired | Nil | 6 | Blood | Strong | FOX-CEF/SUL-CHL-CIP-FEP-CTX-CAZ-ERT-GEN-AMK-MERO-NET-PTZ-TET |
| VB43 | 30 | M | Discharged | Nil | 5 | Blood | negative | FOX-CEF/SUL-CHL-CIP-CPD-FEP-CTX-CAZ-ERT-GEN-AMK-MERO-MIN-NET-PTZ-TET |
| VB44 | 27 | M | Discharged | Nil | 19 | Blood | negative | FOX-CEF/SUL-CHL-CIP-CPD-FEP-CTX-CAZ-ERT-GEN-AMK-MERO-MIN-NET-PTZ-TET |
| VB45 | 56 | F | Discharged | nil | 0 | Blood | Strong | FOX-CEF/SUL-CHL-CIP-CPD-FEP-CTX-CAZ-ERT-GEN-AMK-MERO-MIN-NET-PTZ-TET |
| VB46 | 63 | M | Expired | Nil | 8 | Blood | Strong | FOX-CEF/SUL-CHL-CIP-CPD-FEP-CTX-CAZ-ERT-GEN-AMK-MERO-MIN-NET-PTZ-TET |
| VB47 | 9 | M | Expired | Nil | 3 | Blood | Weak | FOX-CEF/SUL-CHL-CIP-CPD-FEP-CTX-CAZ-ERT-GEN-AMK-MERO-MIN-NET-PTZ-TET |
| VB48 | 28 | F | Expired | Nil | 2 | Blood | Weak | CEF/SUL-CPD-FEP-CTX-CAZ-ERT-GEN-MERO-PTZ |
| VB49 | 60 | F | Expired | Blood pressure, Diabetes mellitus | 0 | Blood | Moderate | FOX-CEF/SUL-CHL-CIP-CPD-FEP-CTX-CAZ-ERT-GEN-AMK-MERO-MIN-NET-PTZ-TET-TIGI |
| VB50 | 51 | M | Discharged | Diabetes mellitus | 12 | Blood | negative | FOX-CEF/SUL-CHL-CIP-CPD-FEP-CTX-CAZ-ERT-GEN-AMK-MERO-MIN-NET-PTZ-TET |
| VB51 | 25 | F | Expired | Haemolytic anaemia | 1 | Blood | Moderate | FOX-CEF/SUL-CHL-CIP-CPD-FEP-CTX-CAZ-ERT-GEN-AMK-MERO-MIN-NET-PTZ-TET |
| VB52 | 64 | M | Expired | Diabetic mellitus | 1 | Blood | Moderate | CIP-CPD-FEP-CTX-CAZ-GEN |
| VB53 | 2 | M | Expired | Nil | 1 | Blood | Moderate | FOX-CFP/SUL-CHL-CIP-CPD-FEP-CTX-CAZ-ERT-GEN-AMK-MERO-MIN-NET-PTZ-TET |
| VB54 | 0 | F | Discharged | Anaemia | 18 | Blood | negative | All susceptible |
| VB55 | 63 | M | Discharged | Diabetic mellitus | 9 | Sputum | negative | CXM-CRO-CAZ-AMK-LEV-LIN-MIN-TEC-TIGI |
| VB56 | 39 | M | Discharged | Stem cell transplantation | 1 | Sputum | negative | CXM-CRO-CAZ-AMK-LEV-LIN-MIN-TEC-TIGI |
| VB57 | 20 | F | Discharged | kidney injury | 9 | Sputum | negative | CAZ-TIGI |
| VB58 | 17 | M | Discharged | liver failure | 9 | Sputum | negative | CXM-CRO-CAZ-LIN-TEC-TIGI |
| VB59 | 25 | M | Discharged | Diabetic insipidus | 11 | Sputum | negative | CRO-CAZ |
| VB60  (C8) | 60 | M | Expired | Diabetic mellitus | 19 | Sputum | Strong | CXM-CRO-CAZ-AMK-LEV-LIN-MIN-TEC-TIGI |
| VB61 | 9M 2D | M | Discharged | Cardiac Failure | 27 | Sputum | Weak | CXM-CRO-CAZ-AMK-LEV-LIN-MIN-TEC-TIGI |
| VB62 | 67 | M | Discharged | Multiple infract state | 13 | Sputum | Weak | CXM-CRO-CAZ-AMK-LEV-LIN-MIN-TEC-TIGI |
| VB63 | 2 | M | Discharged | Esophageal Atresia | 7 | Sputum | negative | CXM-CAZ-AMK-LEV-LIN-MIN-TEC |
| VB64 | 60 | M | Expired | Nil | 4 | Sputum | negative | CAZ-TIGI |
| VB65  (C7) | 49 | M | Expired | Diabetes mellitus | 76 | Sputum | Strong | CXM-CAZ-AMK-LEV-LIN-MIN-TEC |
| VB66 | 75 | M | Discharged | Myocardial infraction | 7 | Sputum | negative | CXM-CAZ-AMK-LIN-MIN-TEC-TIGI |
| VB67 | 33 | M | Discharged | Lung metastasis | 13 | Sputum | negative | All susceptible |
| VB68 | 37 | M | Discharged | Nil | 21 | Sputum | negative | All susceptible |
| VB69 | 47 | M | Discharged | PLHIV-1 | 30 | Sputum | Moderate | All susceptible |
| VB70 | 59 | M | Discharged | Brain injury | 3 | Sputum | Weak | All susceptible |
| VB71 | 19 | M | Discharged | Extradural hematoma | 31 | Sputum | Weak | CXM-CRO-CAZ-AMK-LIN-MIN-TEC-TIGI |
| VB72 | 36 | M | Discharged | Kidney injury | 34 | Sputum | Weak | MIN |
